# MSAP analysis reveals diverse epigenetic statuses in opium poppy varieties with different benzyisoquinoline alkaloid content

**DOI:** 10.3906/biy-1911-69

**Published:** 2020-04-02

**Authors:** Betül BULUT, Zehra AYDINLI, Mine TÜRKTAŞ-ERKEN

**Affiliations:** 1 Department of Biology, Faculty of Science, Çankırı Karatekin University, Çankırı Turkey

**Keywords:** BIA, epigenetics, methylation, MSAP, opium poppy

## Abstract

DNA methylation is one of the major epigenetic modifications influencing the regulation of gene expression. The opium poppy is an important medicinal plant. Its latex contains opium, which is a rich source of pharmaceutical benzyisoquinoline alkaloids (BIA). Here, the methylation-sensitive amplification polymorphism (MSAP) profiling technique using 21 MSAP molecular markers was applied in order to compare levels of DNA methylation between 6 opium poppy varieties. MSAP profiling reflected the different methylation statuses among opium poppy varieties having divergent BIA content. Moreover, different organ-specific epigenetic profiles were observed between the samples. Differential epigenetic profiles of capsules and shoots from the leaves pointed to the impact of methylation on BIA biosynthesis. The data implied that the different DNA methylation status may have important biological significance, in the case of alkaloid content in opium poppy in particular.

## 1. Introduction

*Papaver somniferum *L., commonly known as opium poppy, is one of the most important medicinal plants due to its valuable benzyisoquinoline alkaloids (BIA). The latex of poppy capsule is composed of several BIAs, including morphine, codeine, thebaine, and noscapine (Page, 2005). The concentrations of alkaloids can vary widely among different cultivars of poppy seeds, which is related to the variety of poppy plant, geographical location, and time of harvest as well as processing (Bornhorst and Mbughuni, 2019).

Up to 30 alkaloids have been isolated from *P. somniferum *(Dasgupta, 2019). These alkaloids are the main compounds of opioid analgesics, which are used as effective painkillers in medicine. Today, opium poppy still remains the only commercial source of morphinan alkaloids. Due to these unique features, the genome sequence of the opium poppy has been recently reported (Guo et al., 2018).

The main legal manufacturers of opium poppy under the supervision of the United Nations Organization are Turkey, India, Australia, Spain, France, and Hungary. Turkey and India are the two traditional producers; Turkey has the largest production area in the world, having 13,500 ha total growing area (Gurkok et al., 2015).

Unlike animals, plants stably pass their methylation status on to the next generations. Therefore, the study of epigenetics in plants has been a focus of researchers for a long time. DNA methylation is one of the key epigenetic modifications and is known to play an important role in regulating gene expression during several biological processes (Ci et al., 2015; He et al., 2017; Ngom et al., 2017; Yao et al., 2018). Although the influence of epigenetics on transcriptional control has been widely studied, less is known about its tissue-specificity.

So far, the contribution of epigenetic processes to metabolic diversity has been overlooked. Only a few studies have been dedicated to unveiling the roles of epigenetics in secondary metabolism (Strauss and Reyes-Dominguez, 2011; Kooke et al., 2019). It was found that fungal alkaloid production is regulated by the methylation status of histones H3K9 and H3K27 (Chujo and Scott, 2014). They found that the status of histone H3 lysine 9 and lysine 27 trimethylation (H3K9me3/H3K27me3) at the genes for lolitrem biosynthesis (*LTM*) and ergot alkaloid biosynthesis (*EAS*) are critical regulators of transcriptional activity. On the other hand, there have been no studies conducted on the influence of epigenetics on plant alkaloid content.

The MSAP technique utilizes the restriction isoschizomer pairs *HpaII* and *Msp*I. These enzymes recognize the same restriction site (5’-*CCGG-*3’) but have different sensitivities to certain methylation states of cytosines (Baurens et al., 2003). *Hpa*II is insensitive to full methylation but sensitive to the hemimethylated site of external cytosine, while *Msp*I cuts the internal methylated cytosine. Thus, for a given DNA sample, differential methylated cytosines at the assayed *CCGG* sites can be distinguished using these 2 restriction enzymes. 

To address the question whether opium varieties differing in BIA content also differ epigenetically, we performed MSAP analysis. To further extend our understanding of tissue-specific epigenetic effects in plants, we also analyzed DNA methylation in shoots and capsules. To our knowledge, this is the first study on epigenetic regulations in opium poppy varieties.

## 2. Materials and methods

### 2.1. Plant materials

The seeds of 6 *Papaver somniferum* varieties differing in their benzylisoquinoline alkaloid (BIA) content were provided by Toprak Mahsulleri Ofisi (TMO; Ankara, Turkey). Having 0.6% morphine, <0.02% thebaine, and <0.02% noscapine content, *P. somniferum* cv Ofis-95 and Ofis-96 were used as controls, while *P. somniferum *cv Ofis-1 and Ofis-2 (both with 1.8% morphine content) were used as morphine-rich samples. *P. somniferum *cv TMO-T was used as a thebaine-rich sample (1.8% thebaine content), and *P. somniferum *cv Ofis-NP was used as a noscapine-rich sample (1.29% noscapine content). The seeds were potted in soil and transferred to a growth chamber. Plants were kept with day/night cycles of 16/8 h at 20/24 °C photoperiod, and 4-month-old leaves were collected. The young capsules and shoots of *P. somniferum *cv Ofis-1 and Ofis-2 varieties prior to flowering were also collected. The samples were immediately frozen in liquid nitrogen and stored at –80 °C until analyzed. 

### 2.2. DNA isolation

An E.Z.N.A. HP Plant DNA Mini Kit (Omega Bio-Tek, Norcross, GA, USA) was used for total genomic DNA isolation. The protocol was applied according to the manufacturer’s instructions. The quality and quantity of the DNA samples were assessed by 0.8% agarose electrophoresis and a Nanodrop 2000c spectrophotometer (Thermo Scientific, Vilnius, Lithuania). DNA samples were preserved at –20 °C for use. Three biological replicates were used for each opium poppy sample.

### 2.3. MSAP assay

Methylation-sensitive amplification polymorphism analysis was carried out based on a previous protocol with few modifications (Baurens et al., 2008). The isoschizomers *Hpa *II and *Msp *I were used as frequent-cutter enzymes. *Hpa*II and *Msp*I are methylation-sensitive restriction endonucleases, both recognizing CCGG sequences. *Hpa*II can only cleave nonmethylated CCGG and hemimethylated mCCGG sequences, while *Msp*I can digest nonmethylated CCGG and hemi- or fully methylated CmCGG sequences, but not hemi- and fully methylated mCCGG and mCmCGG sequences (Fulnecek and Kovarik, 2014). The MSAP profiling involved the use of a single preselective primer combination, along with combinations based on 6 *Eco*RI (Eco) and 5 *Hpa*II selective primers (HM) (Table 1). Selective primers were designed to be compatible with the adapter regions with arbitrarily selected two- or three-base extensions.

**Table 1:** List of adapter and primer sequences.

Adapters/Primers	Sequences (5’–3’)
EcoR1 adapter 5	CTCGTAGACTGCGTACC
EcoR1 adapter 3	AATTGGTACGCAGTCTAC
HpaII/MspI adapter 5	GATCATGAGTCCTGCT
HpaII/MspI adapter 3	CGAGCAGGACTCATGA
Eco + 1	GACTGCGTACCAATTCA
HM + 1	ATCATGAGTCCTGCTCGGT
Eco + AG	GACTGCGTACCAATTCAG
Eco + AT	GACTGCGTACCAATTCAT
Eco + AC	GACTGCGTACCAATTCAC
Eco + AAC	GACTGCGTACCAATTCAAC
Eco + ACG	GACTGCGTACCAATTCACG
Eco + ACT	GACTGCGTACCAATTCACT
HM + TAA	ATCATGAGTCCTGCTCGGTAA
HM + TTC	ATCATGAGTCCTGCTCGGTTC
HM + TAG	ATCATGAGTCCTGCTCGGTAG
HM + TGG	ATCATGAGTCCTGCTCGGTGG
HM + TTA	ATCATGAGTCCTGCTCGGTTA

Approximately 500 ng genomic DNA was digested with 10 U *Eco*R1 and 10 U *Msp*I or 20 U *Hpa*II (Thermo Scientific) in a total volume of 25 µL at 37 °C for 22 h. After enzyme inactivation at 80 °C for 20 min, adapters were ligated to digested fragments using T4 ligase (Fermentas) at 23 °C for 4 h. Preamplification and selective amplification were conducted using 1/10 dilutes of ligation products as template. The total volume of 25 μL of preamplification mixture contained: 10 μM Eco + 1 primer, 10 μM HM + 1 primer, 1 × *Taq* buffer, 0.25 mM dNTP, 2 mM MgCl2, 1.5 U DNA Polymerase (Fermentas, Waltham, MA, USA). The PCR conditions were as follows: initial denaturation of 1 min at 94 °C, 30 cycles of 30 s at 94 °C, 30 s at 56 °C, 60 s at 72 °C, final elongation of 10 min at 72 °C. After control of preselective PCR products by visualizing on agarose gel, selective amplification was performed with 21 different combinations of the selective primers. The PCR conditions of selective PCR are as follows: 94 °C, 5 min; 12 times [94 °C, 30 s, 65 °C, 1 min (–0.7 °C per cycle); 72 °C, 1 min]; 23 times [94 °C, 30 s; 56 °C, 1 min; 72 °C, 1 min]; 72 °C, 5 min. The amplicons were resolved by electrophoresis through a 6% denaturing polyacrylamide gel. 

### 2.4. Band scoring

Clearly distinguishable DNA fragments were scored in a form of binary matrix as either present (1) or absent (0) for the samples. All of the identified bands can generally be classified into 4 types, namely, (+, +), (+, −), (−, +), and (−, −). The R package of “*msap*” was used for analysis of binary data (Perez-Figueroa, 2013). The principle coordinates analysis (PCoA) was conducted using the “*msap*” program. The analyses were performed with the samples grouped by their BIA content. The grouping was as follows: control samples, leaves of morphine-rich samples, leaves of thebaine-rich samples, leaves of noscapine-rich samples, shoots of morphine-rich samples, and capsules of morphine-rich samples. The cluster analyses were based on Jaccard’s index (Reif et al., 2005).

## 3. Results

Leaves of 6 opium poppy varieties, together with shoots and capsules of 2 morphine-rich varieties, were analyzed using 21 MSAP molecular markers. MSAP profiling reflected the different methylation status between opium poppy varieties having divergent BIA content. Moreover, tissue-specific epigenetic profiles were observed for the samples.

### 3.1. MSAP fragments

**Figure 1:**
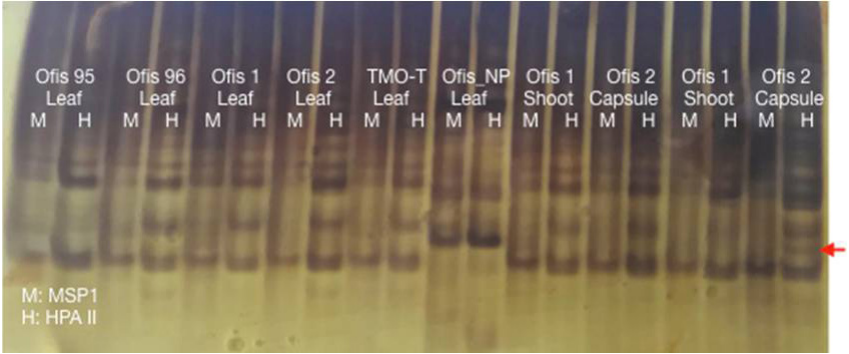
A representative polyacrylamide gel image. The sample names are given on the figure. A different band between the samples is shown as an arrow.

The “*msap*” program, an R package for analyzing MSAP data, was used to estimate the methylation status of the samples. Only the clear bands were used for scoring; a total of 194 bands were scored. A representative gel image is given in Figure 1. Among the scored bands, 164 methylation susceptible loci (MSL) and 30 nonmethylated loci (NML) were distinguished. The number of polymorphic MSL was 130 (79%) and the NML was 12 (40%), indicating a high polymorphism level within the samples. 

To evaluate the relationship between the plant alkaloid content and epigenetic status of the genome, we performed an “*msap*” algorithm by grouping the samples based on their BIA content. Although the overall level of DNA methylation within analyzed *CCGG* loci was comparable between the samples, the methylation profiles showed variations. The MSAP bands that revealed different methylation patterns were divided into 4 types (Table 2). Type I is unmethylation (HPA+/MSP+), type II is hemimethylation (HPA+/MSP–), type III is internal cytosine methylation (HPA–/MSP+), and type IV (HPA–/MSP–) is full methylation. In all groups, type I bands were found to be the most frequent fragments, and types II and III were detected at the lowest levels. The morphine-rich and thebaine-rich varieties possessed similar profiles, while the noscapine-rich sample showed the highest fully-methylated fragments, also revealing the lowest unmethylated fragments, together with the capsule of Ofis-2. Moreover, different methylation profiles have been observed between the organs. Compared to leaves, capsules and shoots had slightly lower methylation levels.

**Table 2:** Levels of 4 DNA methylation patterns of the opium samples classified by BIA content. The levels are given as percentages.

	Leaves ofOfis-95, Ofis-96	Leaves ofOfis-1, Ofis-2	Leaves of TMO-T	Leaves of Ofis-NP	Shoots ofOfis-1, Ofis-2	Capsules of Ofis-1, Ofis-2
Type I (Nonnmethylation)	37.2%	34.1%	34%	29.9%	35.2%	32.6%
Type II (Hemimethylation)	17%	18%	20%	18.3%	17.4%	19.2%
Type III (Internal methylation)	17.7%	18.3%	15.9%	13.4%	20.0%	21.0%
Type IV (Full methylation)	28.1%	29.6%	31.1%	38.4%	27.4%	27.1%

### 3.2. Epigenetic/genetic divergence

**Figure 2:**
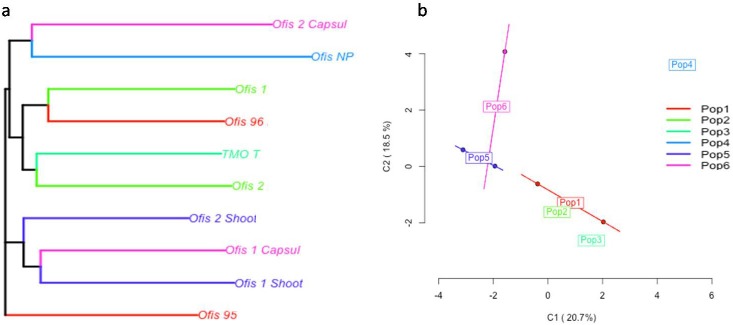
NJ tree (a) and PCoA plot (b) of the samples classified as MSL (methylation-susceptible loci). Pop 1) Leaves of Ofis 95 and Ofis 96; Pop 2) Leaves of Ofis 1 and Ofis 2; Pop 3) Leaves of TMO-T; Pop 4) Leaves of Ofis-NP; Pop 5) Shoots of Ofis 1 and Ofis 2; 6) Leaves of Ofis 1 and Ofis 2.

**Figure 3:**
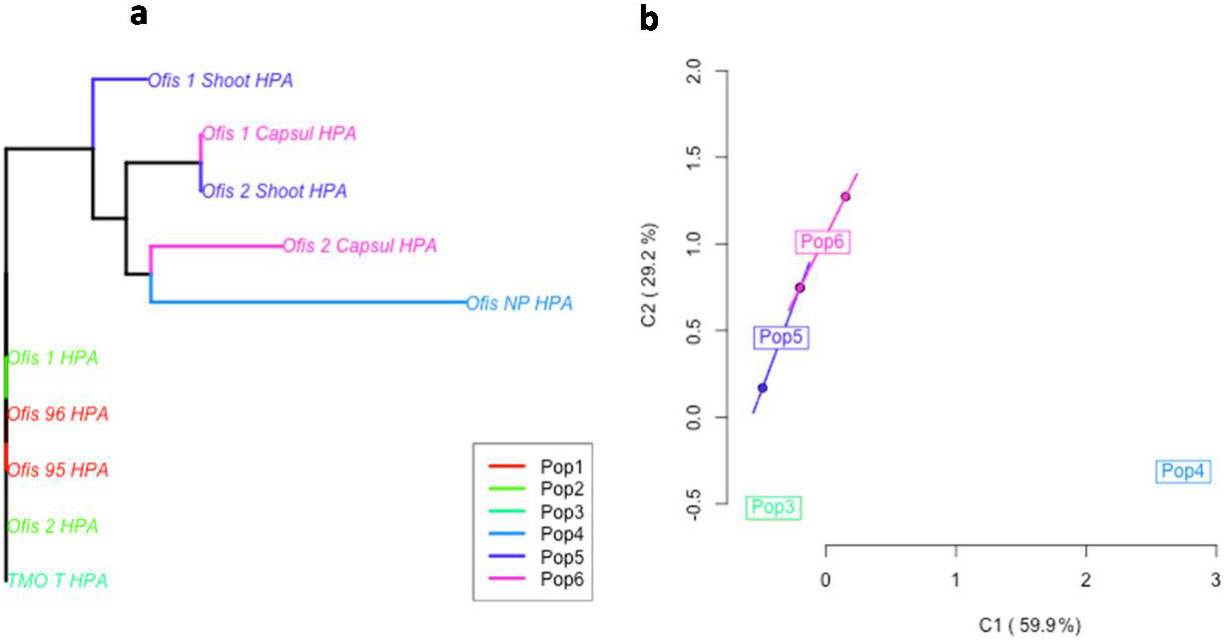
NJ tree (a) and PCoA plot (b) of the samples classified as NML (nonmethylated loci). Pop 1) Leaves of Ofis 95 and Ofis 96; Pop 2) Leaves of Ofis 1 and Ofis 2; Pop 3) Leaves of TMO-T; Pop 4) Leaves of Ofis-NP; Pop 5) Shoots of Ofis 1 and Ofis 2; 6) Leaves of Ofis 1 and Ofis 2.

The MSAP analysis was performed in order to assess both genetic and methylation diversity. The “*msap*” package classifies every locus as either *MSL *(methylation-susceptible loci) or *NML *(non-methylated loci). *MSL*s are used to assess epigenetic variation, whereas *NML*s are used to assess genetic variation. The analysis revealed epigenetic variation between the samples (Figure 2a–b), while minor genetic differences were present between the samples (Figure 3a–b). An organ-specific epigenetic divergence of the samples was also observed in the plot. The capsules and shoots have clearly diverged from the leaves. Additionally, the noscapine-rich sample was placed in a different coordinate in the analysis from the other samples. Interestingly, the capsule of Ofis-2 variety also showed a divergent profile from the other samples. The principle coordinates analysis (PCoA) also supported this differentiation (Figure 2b).

The pattern revealed a trend towards a slight decrease of the methylation within the control plants. PCoA analysis clearly revealed the grouping of the samples based on their different BIA contents (Figure 2b). The thebaine-rich and noscapine-rich samples diverged from the morphine-rich (Ofis1 and Ofis 2) and control samples (Ofis 95 and Ofis 96). Furthermore, an organ-based differentiation was also observed in the analysis. The shoot, capsule, and leaf samples clustered differently from each other, indicating the influence of organ specificity in methylation. 

## 4. Discussion

DNA methylation is involved in several biological processes. In general, plants use epigenetic mechanisms to withstand environmental variations. This is a dynamic process, the differences in which depend on several factors, such as the degree of development and organ type. 

The MSAP technique was performed to profile the methylation status of opium poppy varieties. Although there is extensive variation in the angiosperm, the average methylation level has been found to be about 50% (Niederhuth et al., 2016). Comparing that, the total level of methylation was found to be high in all of the varieties in this study. The slight difference in the frequency of methylated sites between the samples implied that the global level of DNA methylation has little effect on alkaloid content in the analyzed varieties. Similarly, *Secale* taxa, *Populus trichocarpa*, and *T. turgidum* spp. *dicoccoides* possessed a high DNA methylation level (Venetsky et al., 2015; Kalinka et al., 2017; Liang et al., 2019). The authors attribute this situation to the high repeat content of the genomes. On the basis of this consideration, with a rate of 71%, the opium genome is among the more highly repetitive plant genomes (Guo et al., 2018); the observed high methylation status might be a reflection of these regions.

Although the methylation frequencies are comparable between the varieties, the methylation patterns revealed differences among the samples. It is known that the plant alkaloids act as defense compounds (Gurkok et al., 2015; Matsuura and Fett-Neto, 2017). Compared to the control plants, the observed demethylation pattern among the samples with high BIA content might be related to the expression of defense genes that are involved in alkaloid biosynthesis. 

Compared to the other samples, the noscapine-rich sample (Ofis-NP) showed both epigenetic and genetic divergence. During BIA biosynthesis, morphine, thebaine, and noscapine are derived from S-reticuline via different pathways. The conversion of thebaine to morphine occurs through a bifurcated pathway. Thebaine is the precursor of morphine, while synthesis of noscapine proceeds in a completely different branch of the BIA pathway (Beaudoin and Facchini, 2014; Gurkok et al., 2016). Since the samples with different BIA content were epigenetically differentiated, it may be speculated that the difference between the epigenetic status of the samples with different alkaloids may also be a reflection of the differing biosynthetic pathways of these 3 alkaloids. 

It has been found that the methylation status varies greatly between the tissues in *Arabidopsis *(Widman et al., 2014). Organ-specific epigenetic marks contributed to plant propagation in *A. thaliana *(Wibowo et al., 2018). Another study also showed differences in methylation patterns among sorghum tissues (Zhang et al., 2011). The same profile was also identified in maize (Wang et al., 2009). Similarly, an organ-specific methylation pattern was documented in cocoa (Lopez et al., 2010). The researchers found minor genetic differences between the samples, while a clear epigenetic variation was detected between tissues from the same plant. Here, we have also showed that epigenetic changes with minimal genetic differences were accompanied by variation in an organ-specific manner. In our analyses, capsule and shoot samples clearly diverged from the leaves. Differential expression of BIA genes in relation to the organ-specific biosynthesis of the different types of alkaloids has previously been reported (Facchini and De Luca, 1995; Gurkok et al., 2016). It has been proposed that capsules are sites of alkaloid accumulation and stems the sites of alkaloid biosynthesis. Together with these, the differing epigenetic profiles of capsule and shoot from the leaves point to the impact of methylation on BIA biosynthesis.

The capsule of the Ofis-2 variety also showed an epigenetic divergence from the other samples of capsule and shoot. It is known that epigenetic status affects many metabolic and morphological processes. To find an explanation for this result, we compared the morphological characteristics. Since alkaloid content and shoot morphology are comparable between Ofis-1 and Ofis-2 samples, and no relationship between capsule shape and alkaloid content has been detected (Gevenkiris, 2011), we evaluated the morphological features of the capsules. The observation revealed that the shapes of the capsules are different: pear-shaped for Ofis-1 and spherical for Ofis-2. Although it is still an open question, the diversity of Ofis-2’s capsule might be attributed to its morphological difference.

In this study, MSAP technology has been confirmed to be an efficient, reliable detection tool for DNA methylation analysis. We have found that the differing DNA methylation status may have important biological significance, in the case of alkaloid content in particular. 

## Acknowledgments

This study was supported by Çankırı Karatekin University, Bilimsel Araştırma Projeleri Birimi (BAP) (Grant FF061218L04).
